# Imaging Efficacy of [^18^F]CTT1057 PET for the Detection of PSMA-Positive Tumors Using Histopathology as Standard of Truth: Results from the GuideView Phase 2/3 Prospective Multicenter Study

**DOI:** 10.2967/jnumed.124.269007

**Published:** 2025-08

**Authors:** Andrei Iagaru, Jose F. Suarez, Spencer Behr, Rahul Aggarwal, Pilar Paredes, Nicolo Buffi, Thomas Penhoat, Francesco Ceci, Jochen Walz, Nicolas Doumerc, Medge Coulanges, Zhongying Xu, Christelle Seigne, Celine Wilke, Ana M. Catafau, Stefano Fanti, Tobias Maurer, on behalf of the GuideView study investigators

**Affiliations:** 1 Stanford University, Stanford, California;; 2 Department of Urology, Bellvitge University Hospital, IDIBELL, Barcelona, Spain;; 3 University of California San Francisco, San Francisco, California;; 4 Clinic Hospital Barcelona, Institut d’Investigacions Biomèdiques August Pi i Sunyer, University of Barcelona, Barcelona, Spain;; 5 Department of Biomedical Sciences, Humanitas University, Milan, Italy;; 6 Louis Pradel Hospital, Bron, France;; 7 IEO European Institute of Oncology IRCCS, University of Milan, Milan, Italy;; 8 Institut Paoli-Calmettes Cancer Centre, Marseille, France;; 9 Toulouse Hospitals, Toulouse, France;; 10 Advanced Accelerator Applications, a Novartis Company, Geneva, Switzerland;; 11 Novartis Pharma AG, East Hanover, New Jersey;; 12 Novartis Pharma AG, Basel, Switzerland;; 13 Nuclear Medicine, IRCCS AOU di Bologna, Bologna, Italy; and; 14 Department of Urology and Martini-Klinik Prostate Cancer Center, University of Hamburg-Eppendorf, Hamburg, Germany

**Keywords:** high-risk prostate cancer, molecular imaging, PET, prostate-specific membrane antigen, [^18^F]CTT1057

## Abstract

[^18^F]CTT1057 is a highly selective prostate-specific membrane antigen (PSMA)–targeted PET radiotracer for prostate cancer (PCa) detection. This prospective study (GuideView, NCT04838626) evaluates the imaging efficacy of [^18^F]CTT1057 PET to detect PSMA-positive lesions against histopathology in patients with newly diagnosed, untreated, high-risk PCa. **Methods:** Between September 7, 2021, and October 26, 2023, 201 patients planned for radical prostatectomy were screened and 195 patients were enrolled. Of these, 184 patients received a median of 355 MBq (range, 195–400 MBq) of [^18^F]CTT1057 and underwent PET/CT 90 min (±30 min) later. Three masked central independent readers evaluated the images. Coprimary endpoints were patient-level sensitivity (including primary tumor and pelvic lymph nodes) and region-level specificity (including pelvic lymph nodes only) for detection of PSMA-positive lesions, using histopathology as the standard of truth. The lower-bound 95% CI needed to surpass 50% for patient-level sensitivity and 70% for region-level specificity. Success was defined as at least 2 of 3 central readers meeting these criteria. Secondary endpoints included the patient-level and region-level positive predictive value and accuracy, region-level sensitivity, inter- and intrareader variability, detection rate of distant metastasis, pharmacokinetics, and safety and tolerability assessments. **Results:** Of the 184 patients who received [^18^F]CTT1057, 172 patients were evaluable for efficacy. Among these, a median of 19 lymph nodes (interquartile range, 13.0–28.5 lymph nodes) were dissected per patient. Both coprimary endpoints were met, with lower bounds of 95% CIs surpassing the success criteria for all 3 readers for both patient-level sensitivity (range, 86.8%–90.0%; lower-bound 95% CI, 80.7%–84.5%) and region-level specificity (97.1%; lower-bound 95% CI, 92.7%). Interreader variability Fleiss κ was 63.9%; intrareader reproducibility Cohen κ was 89.4%–100%. [^18^F]CTT1057 had a favorable safety profile. **Conclusion:** GuideView confirmed the imaging efficacy of [^18^F]CTT1057 for the detection of PSMA-positive lesions, with high patient-level sensitivity and region-level specificity. Substantial interreader variability and almost perfect intrareader reproducibility suggest that [^18^F]CTT1057 findings are robust and reliable. [^18^F]CTT1057 will contribute to expanding access to PSMA PET imaging to properly diagnose and treat patients with PCa.

## Visual Abstract

**Figure:**
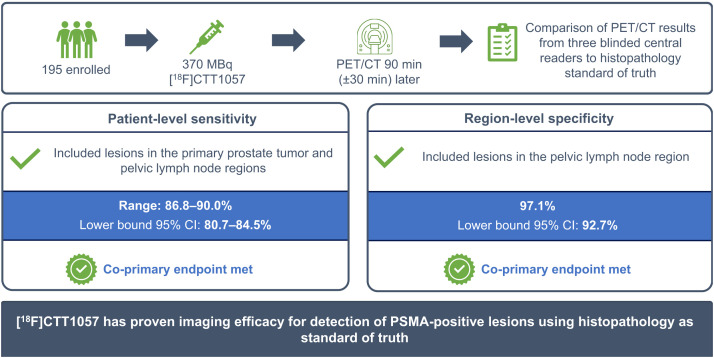

Imaging of prostate cancer (PCa) has been transformed over the last 10 y through the development of novel prostate-specific membrane antigen (PSMA)–targeted PET radiotracers, which have shown enhanced sensitivity and specificity for PCa detection compared with conventional imaging techniques (bone scintigraphy, CT, and MRI) (1). The latest National Comprehensive Cancer Network Clinical Practice Guidelines in Oncology for PCa recommend PSMA PET as an effective frontline imaging technique for the detection of primary PCa lesions (2). [^68^Ga]Ga-PSMA-11 was the first PSMA PET radiotracer approved for primary staging of patients with high-risk PCa and restaging of patients with biochemical recurrence of PCa (3), followed by ^18^F-labeled PET radiotracers, including [^18^F]DCFPyL (4*–*6), [^18^F]rhPSMA-7.3 (7*,*^8^), and [^18^F]PSMA-1007 (9*,*^10^). ^18^F-labeled radiotracers offer the benefits of more scalable batch production than ^68^Ga-labeled radiotracers, and their longer half-life enables delayed PET acquisition (11). Imaging with PSMA-targeted PET radiotracers also helps select patients who may benefit from radiopharmaceutical therapy, such as [^177^Lu]Lu-PSMA-617. However, wider availability of effective and accurate PET radiotracers is required, because patient access to these imaging modalities is inadequate in certain geographical regions.

The PSMA-targeted ^18^F-labeled PET radiotracer [^18^F]CTT1057 (vidoflufolastat [^18^F]) contains a phosphoramidate scaffold and has been shown to pseudoirreversibly bind to PSMA with high nanomolar affinity (12), which might account for a high tumor-to-background ratio and prolonged tumor uptake (13). GuideView (NCT04838626) aimed to evaluate the safety and imaging efficacy of [^18^F]CTT1057 for the detection of PSMA-positive tumors, using histopathology as the standard of truth in patients with primary PCa planned for surgical therapy. To our knowledge, this is the first study that used both histopathology of the primary tumor (PT) and pelvic lymph nodes (PLNs) to assess the imaging efficacy of a PSMA PET radiotracer.

## MATERIALS AND METHODS

### Patients

GuideView was a multicenter, single-arm, open-label prospective study of patients with newly diagnosed, untreated, high-risk PCa according to the D’Amico classification (defined as clinical stage ≥ T2c and/or prostate-specific antigen level > 20 ng/mL, and/or Gleason score ≥ 8) (14) for whom radical prostatectomy with extended PLN dissection was planned (NCT04838626; Supplemental Table 1 gives full inclusion and exclusion criteria [supplemental materials are available at http://jnm.snmjournals.org]) (1). The protocol was approved by the institutional review board at each study site; all patients gave written informed consent. GuideView was undertaken in accordance with the Declaration of Helsinki and the International Council for Harmonisation of Technical Requirements for Pharmaceuticals for Human Use E6 Guideline for Good Clinical Practice.

Between September 7, 2021, and October 26, 2023, 201 patients were screened across 16 sites in Europe and 1 site in the United States; 195 patients were enrolled (Full Analysis Set), and 184 patients received [^18^F]CTT1057 (Safety Analysis Set; Supplemental Fig. 1). The Efficacy Analysis Set, defined as all enrolled patients who received a dose of [^18^F]CTT1057, with both an evaluable [^18^F]CTT1057 PET/CT scan and a histopathology assessment, and who had not received prohibited systemic antineoplastic therapy before the completion of PET/CT and surgery (supplemental materials), contained 172 patients.

### Imaging with [^18^F]CTT1057

Patients received a single intravenous injection of 370 MBq (median, 355 MBq; range, 195–400 MBq) of [^18^F]CTT1057; PET/CT was planned to be performed from the mid-thighs to the vertex 90 min (±30 min) after injection.

Three independent central readers (masked to all clinical information) at the designated contract research organization assessed the [^18^F]CTT1057 PET/CT images. [^18^F]CTT1057-positive PT and PLNs were visually identified as focal uptake higher than physiologic background activity of the anatomic site or blood pool (15*,*^16^).

### Standard of Truth

Radical prostatectomy (prostate gland and local invasion of adjacent structures) and extended PLN dissection surgery were planned between 48 h and 6 wk after [^18^F]CTT1057 PET/CT, with at least 12 PLNs planned to be dissected per patient to ensure adequate representation of positive (i.e., metastatic) and negative (i.e., normal) PLNs. Local pathologists (masked to the [^18^F]CTT1057 PET/CT result) assessed the tissue specimens.

### Efficacy Outcomes

Efficacy outcomes were based on the central review of [^18^F]CTT1057 PET/CT. Patient-level analyses included PT and PLNs; region-level analyses included only PLNs. Patient-level PET scans were judged as positive if at least 1 lesion in the PT or PLN region was [^18^F]CTT1057-positive; a region was considered positive if at least 1 lesion in the PLN was [^18^F]CTT1057-positive.

The coprimary endpoints were patient-level sensitivity and region-level specificity of [^18^F]CTT1057. The supplemental materials detail the secondary and exploratory endpoints.

### Statistical Analyses

For study success, the lower-bound 95% CI for at least 2 of the 3 central readers needed to surpass 70% for region-level specificity and 50% for patient-level sensitivity. Additional statistical analyses are detailed in the supplemental materials.

## RESULTS

Table 1 presents the patient characteristics, [^18^F]CTT1057 dosing, and PET acquisition time. In the efficacy analysis set, a median of 19 lymph nodes (interquartile range, 13.0–28.5 lymph nodes) were dissected per patient; fewer than 12 lymph nodes were dissected from 33 (19.2%) patients.

**TABLE 1.** Patient Characteristics, [^18^F]CTT1057 Dosing, and PET Acquisition Time

Characteristic	Full Analysis Set (*n* = 195)	Efficacy Analysis Set (*n* = 172)
Age (y)	65.0 (61.0–69.0)	65.0 (60.0–69.0)
Race		
White	192 (98.5)	170 (98.8)
Asian	1 (0.5)	0 (0.0)
Unknown	2 (1.0)	2 (1.2)
Ethnicity		
Hispanic or Latino	56 (28.7)	51 (29.7)
Not Hispanic or Latino	122 (62.6)	106 (61.6)
Not reported or unknown	17 (8.7)	15 (8.7)
Body mass index (mg/m^2^)	27.1 (24.5–29.4)	27.2 (24.8–30.0)
Eastern Cooperative Oncology Group performance status		
0	193 (99.0)	170 (98.8)
1	2 (1.0)	2 (1.2)
PSA level at screening (ng/mL)*		
*n*	187	166
Median	11.3 (7.2–21.1)	11.3 (7.7–20.7)
PT clinical stage		
≤T2c	129 (66.2)	116 (67.4)
T3^†^	18 (9.2)	17 (9.9)
T3a	32 (16.4)	29 (16.9)
T3b	11 (5.6)	7 (4.1)
T4	1 (0.5)	1 (0.6)
Tx^‡^ or missing	4 (2.1)	2 (1.2)
Biopsy predominant histology/cytology		
Acinar adenocarcinoma	189 (96.9)	166 (96.5)
Ductal adenocarcinoma	3 (1.5)	3 (1.7)
Other^§^	3 (1.5)	3 (1.7)
Biopsy Gleason score		
≤6	5 (2.6)	5 (2.9)
7 (3 + 4)	29 (14.9)	24 (14.0)
7 (4 + 3)	29 (14.9)	25 (14.5)
8	86 (44.1)	78 (45.3)
9 or 10	46 (23.6)	40 (23.3)
Number of patients with dissected lymph nodes	175	172
Number of lymph nodes dissected per patient	19.0 (13.0–29.0)	19.0 (13.0–28.5)
Number of patients with PLN metastases per histopathology assessment	37 (21.1)	35 (20.3)
Number of patients with PLN metastases ≤ 2 mm	17 (9.7)	16 (9.3)
Time from injection to full-body PET (min)		
*n*	184	172
Median	90.0 (85.0–91.0)	90.0 (84.0–91.0)
Time from injection to additional pelvic PET imaging (min)		
*n*	42	40
Median	125.0 (119.0–139.0)	125.0 (118.5–138.0)
Time from full-body PET imaging to surgery (d)		
*n*	175	172
Median	24.0 (14.0–37.0)	24.0 (14.0–36.5)
Administered radioactivity (MBq)		
*n*	184	172
Median	355.2 (330.0–368.2)	355.2 (329.1–368.5)

* From central laboratory measurements.

† Patients who had T3 PT clinical staging in whom T3a or T3b were not specified.

‡ Clinical stage for PT is captured as per definitions in American Joint Committee on Cancer guidelines for PCa, where Tx means PT cannot be assessed. Clinical staging at initial diagnosis was captured from digital rectal examination.

§ Adenocarcinoma not otherwise specified for all 3 patients.

PSA = prostate-specific antigen.

Continuous data are median with interquartile range in parentheses. Qualitative data are number with percentage in parentheses.

### Coprimary Endpoints

Both coprimary endpoints were met, with the lower-bound 95% CI exceeding the predefined criteria for patient-level sensitivity (86.8%–90.0%; lower-bound 95% CI, 80.7%–84.5%) and region-level specificity (97.1%; lower-bound 95% CI, 92.7%) for all 3 readers (Table 2). No candidate covariates (Gleason score, prostate-specific antigen level at screening, and number of dissected PLNs) significantly affected patient-level sensitivity or region-level specificity (Supplemental Tables 2 and 3). Representative images show high [^18^F]CTT1057 uptake in PT of patients with different Gleason scores (Fig. 1) and true-positive PT and PLN lesions detected by [^18^F]CTT1057 PET/CT (Fig. 2). Delayed pelvic PET/CT images (120–180 min after injection) showed prolonged [^18^F]CTT1057 uptake (Supplemental Fig. 2).

**TABLE 2.** Patient-Level Sensitivity and Region-Level Specificity for Identification of PSMA-Positive PT and/or PLN Lesions in the Efficacy Analysis Set

Parameter	Central reader 1 (*n* = 172)	Central reader 2 (*n* = 172)	Central reader 3 (*n* = 172)
Patient-level sensitivity for detection of PT and/or PLN metastases			
True-positive	145	153	146
False-positive	4	2	3
False-negative	22	17	22
True-negative	1	0	1
Patient-level sensitivity*	86.8 (80.7–91.6)	90.0 (84.5–94.1)	86.9 (80.9–91.6)
Region-level specificity for detection of PLN metastases			
True-positive	7	7	8
False-positive	4	4	4
False-negative	27	27	26
True-negative	134	134	134
Region-level specificity*	97.1 (92.7–99.2)	97.1 (92.7–99.2)	97.1 (92.7–99.2)

* Data are percentage with 95% CI in parentheses.

**FIGURE 1.**
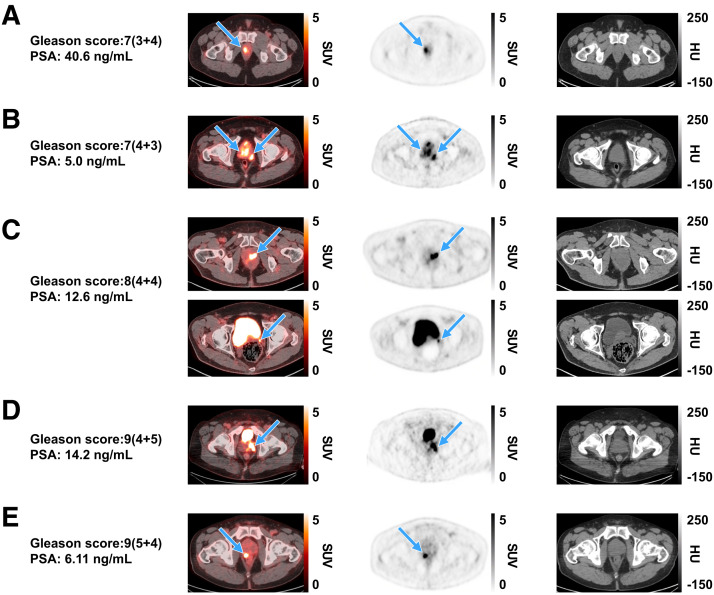
Representative [^18^F]CTT1057 PET/CT scan cases showing true-positive lesions in PT among patients with different Gleason scores (arrows), with SUV_max_ ranges for each across 3 readers. Axial slices (left to right) show fused PET/CT scan, PET scan, and CT scan. (A) A 48-y-old patient (cT3a) with right prostate lesion (SUV_max_, 10.1–12.8). (B) A 63-y-old patient (cT3a) with multiple prostate lesions (SUV_max_, 6.8–10.6). (C) A 69-y-old patient (cT2a) showing left prostate lesion (SUV_max_, 9.6–46.4; top row) with extension to left seminal vesicle (bottom row). (D) A 63-y-old patient (cT3) with left prostate lesion (SUV_max_, 8.7–14). (E) A 77-y-old patient (cT2c) with right prostate lesion (SUV_max_, 12.5–12.7). cT = clinical stage for PT; HU = Hounsfield units; PSA = prostate-specific antigen.

**FIGURE 2.**
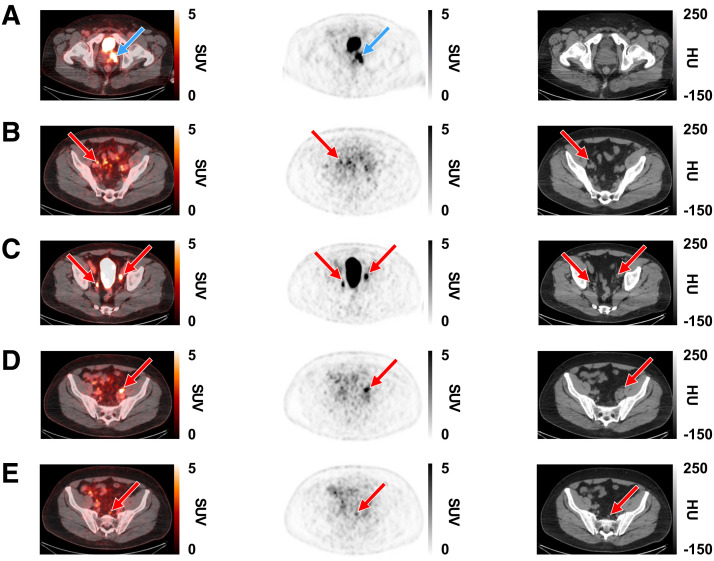
Representative [^18^F]CTT1057 PET/CT scan case showing true-positive lesions in PT and PLN metastases in 63-y-old patient (PT clinical stage 3; Gleason score, 9 [4 + 5]; PSA, 14.2 ng/mL). Axial slices (left to right) show fused PET/CT scan, PET scan, and CT scan. (A) True-positive lesions in left prostate gland (SUV_max_, 14; blue arrows). (B–E) True-positive lesions were seen in several PLNs (red arrows). (B) Right external iliac (SUV_max_, 4.7). (C) Right internal iliac (SUV_max_, 6.9) and left external iliac (SUV_max_, 11.4). (D) Left external iliac (SUV_max_, 10.2). (E) Presacral (SUV_max_, 3.6). HU = Hounsfield units.

### Region-Level Sensitivity

Overall region-level sensitivity ranged from 20.6% to 23.5%. Region-level sensitivity excluding PLN metastases of at least 2 mm in diameter was 26.9%–30.8% (Supplemental Table 4); results were identical for the per-protocol analysis excluding PLN metastases of less than 2 mm.

### Positive Predictive Value, Negative Predictive Value, and Accuracy

Table 3 describes the region-level positive predictive value, negative predictive value, and accuracy. Supplemental Table 5 details the patient-level positive predictive value and accuracy.

**TABLE 3.** Region-Level Positive Predictive Value, Negative Predictive Value, and Accuracy for Identification of PSMA-Positive PLN Lesions in the Efficacy Analysis Set

Parameter	Central reader 1 (*n* = 172)	Central reader 2 (*n* = 172)	Central reader 3 (*n* = 172)
Positive predictive value	63.6 (30.8–89.1)	63.6 (30.8–89.1)	66.7 (34.9–90.1)
Negative predictive value	83.2 (76.6–88.7)	83.2 (76.6–88.7)	83.8 (77.1–89.1)
Accuracy	82.0 (75.4–87.4)	82.0 (75.4–87.4)	82.6 (76.1–87.9)

Data are percentage with 95% CI in parentheses.

### Reader Variability

Interreader variability Fleiss κ for all regions together (whole-body scan/overall regions) was 63.9% (Supplemental Table 6). All scans were agreed upon by at least 2 readers; 163 (88.6%) scans were agreed upon by all 3 readers. Intrareader reproducibility Cohen κ was 89.4%–100% (Supplemental Table 6).

### Detection of Distant Metastasis

Across the 3 central readers, (minimum–maximum) 1–3 distant PSMA-positive lesions (extra-PLN, visceral, or skeletal) were documented on [^18^F]CTT1057 PET/CT in 6–10 (3.3%–5.4%) patients in the safety analysis set (Supplemental Table 7). In total, 10–13 distant PSMA-positive lesions were detected across readers, with 7–9 (53.8%–90.0%), 1–6 (10.0%–46.2%), and 0–1 (0%–8.3%) lesions in the extra-PLN, skeletal, and visceral regions, respectively (Supplemental Table 8). Histopathology was not available for these lesions.

### Quantitative Assessment of [^18^F]CTT1057 Uptake

High [^18^F]CTT1057 uptake (mean SUV_max_ and mean SUV_max _lesion-to-background ratio, respectively) across readers was seen in PT (11.81–15.81 and 4.26–5.75), PLN lesions (5.12–6.16 and 3.82–3.96), and extra-PLN lesions (7.20–8.70 and 4.80–6.08) but was variable in skeletal lesions (2.00–3.08 and 5.00–7.27) and visceral lesions (11.30–not evaluable and 3.90–not evaluable; Supplemental Fig. 3).

### Change in Intended Treatment

After [^18^F]CTT1057 PET/CT, 6 patients (3.3%) had a change in the intended treatment plan from initially planned surgery to radiation plus androgen deprivation therapy (5 patients; Supplemental Fig. 4) or androgen deprivation therapy alone (1 patient; Supplemental Table 9).

### Pharmacokinetics, Metabolism, and Safety

Pharmacokinetic and metabolism assessments were performed for 10 patients at 1 site. The maximum blood radioactivity concentration (mean ± SD, 49.0 ± 19.2 kBq/mL) was observed at the first postdose time point (0–5 min after injection) in all patients (Supplemental Fig. 5). Radioactivity excreted via urine increased over time, with an excreted cumulative percentage (mean ± SD) of injected activity of 37.0% ± 21.4% at the last time point (3–5 h after injection; Supplemental Table 10). Most excreted radioactivity was due to the parent compound; no significant amount of radioactive metabolites was found. The relative contribution (mean ± SD) of the parent compound to the total radioactivity in urine decreased slightly with time, from 96.8% ± 2.3% at the interval from injection to image acquisition starting time to 87.9% ± 6.9% from 3 to 5 h after injection (Supplemental Table 11). The estimated [^18^F]CTT1057-modeled terminal and effective half-life were (geometric mean ± coefficient of variation) 1.84 h ± 18.41% and 0.92 h ± 9.08%, respectively.

[^18^F]CTT1057 had a favorable tolerability and safety profile, with no deaths or fatal events. In the safety analysis set, 24 (13%) patients experienced at least 1 adverse event; the most frequent were hypertension (2.7%), dizziness (1.1%), and presyncope (1.1%). Adverse events suspected to be related to [^18^F]CTT1057 were reported by 1 patient (feeling hot and headache). Two patients (1.1%) experienced a serious adverse event (1 with sepsis syndrome and 1 with myocardial ischemia), although neither were attributed to [^18^F]CTT1057.

## DISCUSSION

GuideView assessed the imaging efficacy of [^18^F]CTT1057 PET/CT for the detection of PSMA-positive tumor lesions using histopathology as the standard of truth in patients with newly diagnosed high-risk PCa. This study met both coprimary endpoints, with predefined success criteria surpassed by all 3 readers.

The region-level specificity endpoint in GuideView included only PLN—consistent with the patient-level specificity endpoint in trials for approved ^18^F-labeled radiotracers indicated for primary staging, [^18^F]DCFPyL (OSPREY study) (5) and [^18^F]rh-PSMA-7.3 (LIGHTHOUSE study) (7)—thereby allowing direct comparison to these trials. Hence, region-level specificity data in GuideView were consistent with patient-level specificity results for [^18^F]DCFPyL (cohort A, 96.3%–98.9%) (5) and [^18^F]rh-PSMA-7.3 (93%–97%) (7), demonstrating comparable imaging efficacy of [^18^F]CTT1057 to approved ^18^F-labeled radiotracers. The high region-level specificity in GuideView supports the clinical suitability of [^18^F]CTT1057 for detecting PLN metastases and could enable tailored therapeutic approach planning.

Patient-level sensitivity also met predefined success criteria in GuideView. Because metastatic PLNs have low prevalence and high variability (4%–58%) in patients with newly diagnosed PCa (17), both PT and PLNs were included in patient-level assessments. Therefore, this endpoint cannot be directly compared with existing PSMA PET radiotracers, because these studies included only PLNs in their patient-level sensitivity analyses (5*,*^7^*,*^18^). To account for inclusion of PT in addition to PLNs, the predefined lower-bound 95% CI threshold was set higher than in previous studies (5*,*^7^). PT is an adequate tissue sample for evaluating the binding of the imaging agent to the target, providing proof of mechanism and thus allowing proper characterization of [^18^F]CTT1057 as a PSMA-targeted molecular PET imaging agent for detection of PSMA-positive tumors (19). Because PSMA-negative PT may indicate a more aggressive variant, assessment of PT using PSMA PET is of clinical relevance (20*,*^21^).

Micrometastases detected on histopathology cannot be easily detected by PET because of the fundamental resolution limits (22*,*^23^). In other PSMA PET studies, increased sensitivity was reported when excluding lesions of less than 3 mm (24) or less than 5 mm in size (5). In a post hoc analysis excluding micrometastases within the defined size limit in the literature (≤2.0 mm), region-level sensitivity in GuideView was comparable to patient-level sensitivity (PLNs only) in studies of [^18^F]DCFPyL (28%–39%) (25) and [^18^F]rhPSMA-7.3 (23%–30%) (7).

GuideView reported substantial interreader variability and almost perfect intrareader reproducibility (26), demonstrating the consistency of [^18^F]CTT1057 PET scan reading, and these results were in line with results reported in the OSPREY study (cohort A, interreader variability, 78%; intrareader reproducibility, 79%–100%) (5). Agreement of at least 2 readers was achieved for all scans; individual values for each reader for each coprimary endpoint were high and consistent. The region-level positive predictive value was broadly high across readers, similar to results reported in OSPREY (cohort A; positive predictive value, 72%–81%) (25) and LIGHTHOUSE (positive predictive value, 57%–70%) (7).

Well-informed treatment planning relies on the accurate identification of distant metastatic disease at initial PCa diagnosis (27). Up to 25% of primary lymphatic landing sites have been found to lie outside the boundaries of an extended PLN dissection (28). In GuideView, PSMA-positive lesions outside of the dissected PT and PLN region were detected in 3.3%–5.4% of patients, mainly in the extra-PLN region. Thus, [^18^F]CTT1057 may help improve visualization of disease location and extent, addressing the unfulfilled medical need for noninvasive imaging techniques for metastatic lesion detection, even at an early stage of the disease.

Limitations include that no pathology assessment or follow-ups were performed on PSMA-positive lesions found outside of the dissected PT and PLN regions; therefore, it could not be confirmed whether these were true metastatic sites. Furthermore, fewer than 12 lymph nodes were removed from a considerable proportion of patients, which may have lowered the false-negative rate. Strengths were that high uptake of [^18^F]CTT1057 was seen in PT even at lower Gleason scores and that [^18^F]CTT1057 had a favorable safety profile, consistent with a phase 1 trial (13).

## CONCLUSION

GuideView provides evidence of the efficacy of [^18^F]CTT1057 PET/CT as a molecular imaging biomarker for the detection of PSMA-positive lesions against histopathology in patients with PCa before initial curative therapy. Both coprimary endpoints were met, supporting that [^18^F]CTT1057 can be added to the armamentarium of PSMA-targeted PET tracers, thus increasing patient access to proper diagnosis and tailored PCa treatment.

## DISCLOSURE

This study was funded by Novartis. Andrei Iagaru’s contribution to this publication was not part of his Stanford University duties or institutional responsibilities. He reports scientific advisory board fees from Alpha9Tx, Clarity Pharmaceuticals, and Radionetics Oncology; research grants from GE HealthCare and Novartis; consulting fees from GE HealthCare, Novartis, Progenics Pharmaceuticals, and Telix; and roles on scientific steering committees for Novartis. Jose Suarez reports honoraria from Astellas, Bayer, Ipsen, and Jansen. Spencer Behr reports honoraria from Novartis. Rahul Aggarwal reports consulting fees and researching funding to his institution from Novartis. Pilar Paredes reports speaker fees and honoraria for advisory boards from Advanced Accelerator Applications, a Novartis company; Astellas; and Bayer. Francesco Ceci reports speaker fees from Bayer, Curium, Janssen Oncology, and Novartis; consultation fees or advisory board fees from Curium, Novartis, and Telix; and research funding from GE HealthCare. Jochen Walz reports honoraria from Intuitive, Lightpoint, Novartis, and Telix. Ana Catafau and Medge Coulanges are employees of Advanced Accelerator Applications, a Novartis company. Celine Wilke, Christelle Seigne, and Zhongying Xu are employees of Novartis. Stefano Fanti reports speaker fees and honoraria for advisory boards from Advanced Accelerator Applications, Amgen, Astellas, Bayer, Blue Earth Diagnostics, Novartis, Telix, and United. Tobias Maurer reports speaker fees from ABX, Astellas, Bayer, Sanofi-Aventis, and Phillips; consultant fees from ABX, Advanced Accelerator Applications International S.A., Ascenian, Astellas, Axiom, Blue Earth Diagnostics, GEMoAb, Novartis, ROTOP Pharma, and Telix; and research funding from ABX, Brainlab, Intuitive Surgical, and Telix. NCCN makes no warranties of any kind whatsoever regarding their content, use or application and disclaims any responsibility for their application or use in any way. No other potential conflict of interest relevant to this article was reported.

## Notes

KEY POINTS **QUESTION:** Does [^18^F]CTT1057 PET/CT provide adequate imaging efficacy to detect PSMA-expressing lesions using histopathology as a standard of truth? **PERTINENT FINDINGS:** In this prospective study, 195 patients were enrolled and 172 patients were evaluable. Both coprimary endpoints were met, with high and consistent patient-level sensitivity (range, 86.8%–90.0%; lower-bound 95% CI, 80.7%–84.5%) and region-level specificity (97.1%; lower-bound 95% CI, 92.7%). **IMPLICATIONS FOR PATIENT CARE:** [^18^F]CTT1057 can be added to the armamentarium of PSMA-targeted PET tracers, thus increasing patient access to proper diagnosis and tailored PCa treatment.

## References

[1] Cornford P van den Bergh RCN Briers E. EAU-EANM-ESTRO-ESUR-ISUP-SIOG guidelines on prostate cancer: 2024 update. Part I: screening, diagnosis, and local treatment with curative intent. Eur Urol. 2024;86:148–163. 38614820 10.1016/j.eururo.2024.03.027

[2] NCCN Clinical Practice Guidelines in Oncology (NCCN Guidelines) for Prostate Cancer V.2.2025. National Comprehensive Cancer Network website. https://www.nccn.org/guidelines/guidelines-detail?id=1459. Accessed May 27, 2025.

[3] Drug approval package: gallium Ga 68 PSMA-11. Food and Drug Administration website. https://www.accessdata.fda.gov/drugsatfda_docs/nda/2020/212642Orig1s000TOC.cfm. Published December 1, 2020. Accessed March 5, 2025.

[4] Morris MJ Rowe SP Gorin MA.; CONDOR Study Group. Diagnostic performance of ^18^F-DCFPyL-PET/CT in men with biochemically recurrent prostate cancer: results from the CONDOR phase III, multicenter study. Clin Cancer Res. 2021;27:3674–3682. 33622706 10.1158/1078-0432.CCR-20-4573 PMC8382991

[5] Pienta KJ Gorin MA Rowe SP. A phase 2/3 prospective multicenter study of the diagnostic accuracy of prostate specific membrane antigen PET/CT with ^18^F-DCFPyL in prostate cancer patients (OSPREY). J Urol. 2021;206:52–61. 33634707 10.1097/JU.0000000000001698 PMC8556578

[6] Metser U Zukotynski K Mak V. Effect of ^18^F-DCFPyL PET/CT on the management of patients with recurrent prostate cancer: results of a prospective multicenter registry trial. Radiology. 2022;303:414–422. 35076300 10.1148/radiol.211824

[7] Surasi DS Eiber M Maurer T.; LIGHTHOUSE Study Group. Diagnostic performance and safety of positron emission tomography with ^18^F-rhPSMA-7.3 in patients with newly diagnosed unfavourable intermediate- to very-high-risk prostate cancer: results from a phase 3, prospective, multicentre study (LIGHTHOUSE). Eur Urol. 2023;84:361–370. 37414702 10.1016/j.eururo.2023.06.018

[8] Jani AB Ravizzini GC Gartrell BA.; SPOTLIGHT Study Group. Diagnostic performance and safety of ^18^F-rhPSMA-7.3 positron emission tomography in men with suspected prostate cancer recurrence: results from a phase 3, prospective, multicenter study (SPOTLIGHT). J Urol. 2023;210:299–311. 10.1097/JU.0000000000003493 PMC12721651 37126069

[9] Exterkate L Hermsen R Küsters-Vandevelde HVN. Head-to-head comparison of ^18^F-PSMA-1007 positron emission tomography/computed tomography and multiparametric magnetic resonance imaging with whole-mount histopathology as reference in localisation and staging of primary prostate cancer. Eur Urol Oncol. 2023;6:574–581. 37230883 10.1016/j.euo.2023.04.006

[10] Mookerji N Pfanner T Hui A. Fluorine-18 prostate-specific membrane antigen-1007 PET/CT vs multiparametric MRI for locoregional staging of prostate cancer. JAMA Oncol. 2024;10:1097–1103. 38949926 10.1001/jamaoncol.2024.3196 PMC11217889

[11] Kesch C Kratochwil C Mier W Kopka K Giesel FL. Gallium-68 or fluorine-18 for prostate cancer imaging? J Nucl Med. 2017;58:687–688. 28408526 10.2967/jnumed.117.190157

[12] Ganguly T Dannoon S Hopkins MR. A high-affinity [^18^F]-labeled phosphoramidate peptidomimetic PSMA-targeted inhibitor for PET imaging of prostate cancer. Nucl Med Biol. 2015;42:780–787. 26169882 10.1016/j.nucmedbio.2015.06.003 PMC4624265

[13] Behr SC Aggarwal R VanBrocklin HF. Phase I study of CTT1057, an ^18^F-labeled imaging agent with phosphoramidate core targeting prostate-specific membrane antigen in prostate cancer. J Nucl Med. 2019;60:910–916. 30464040 10.2967/jnumed.118.220715 PMC6604687

[14] D’Amico AV Whittington R Malkowicz SB. Biochemical outcome after radical prostatectomy, external beam radiation therapy, or interstitial radiation therapy for clinically localized prostate cancer. JAMA. 1998;280:969–974. 9749478 10.1001/jama.280.11.969

[15] Fanti S Minozzi S Morigi JJ. Development of standardized image interpretation for ^68^Ga-PSMA PET/CT to detect prostate cancer recurrent lesions. Eur J Nucl Med Mol Imaging. 2017;44:1622–1635. 28536833 10.1007/s00259-017-3725-1

[16] Fendler WP Calais J Eiber M. Assessment of ^68^Ga-PSMA-11 PET accuracy in localizing recurrent prostate cancer: a prospective single-arm clinical trial. JAMA Oncol. 2019;5:856–863. 30920593 10.1001/jamaoncol.2019.0096 PMC6567829

[17] Petersen LJ Zacho HD. PSMA PET for primary lymph node staging of intermediate and high-risk prostate cancer: an expedited systematic review. Cancer Imaging. 2020;20:10. 31973751 10.1186/s40644-020-0290-9 PMC6979382

[18] Hofman MS Lawrentschuk N Francis RJ.; proPSMA Study Group Collaborators. Prostate-specific membrane antigen PET-CT in patients with high-risk prostate cancer before curative-intent surgery or radiotherapy (proPSMA): a prospective, randomised, multicentre study. Lancet. 2020;395:1208–1216. 32209449 10.1016/S0140-6736(20)30314-7

[19] Sellmyer MA Lee IK Mankoff DA. Building the bridge: molecular imaging biomarkers for 21st century cancer therapies. J Nucl Med. 2021;62:1672–1676. 34446450 10.2967/jnumed.121.262484 PMC8612205

[20] Bakht MK Derecichei I Li Y. Neuroendocrine differentiation of prostate cancer leads to PSMA suppression. Endocr Relat Cancer. 2018;26:131–146. 30400059 10.1530/ERC-18-0226

[21] Rosenzweig B Haramaty R Davidson T. Very low prostate PET/CT PSMA uptake may be misleading in staging radical prostatectomy candidates. J Pers Med. 2022;12:410. 35743645 10.3390/jpm12060861 PMC9225530

[22] van Leeuwen PJ Emmett L Ho B. Prospective evaluation of ^68^gallium-prostate-specific membrane antigen positron emission tomography/computed tomography for preoperative lymph node staging in prostate cancer. BJU Int. 2017;119:209–215. 27207581 10.1111/bju.13540

[23] Kolthammer JA Su KH Grover A Narayanan M Jordan DW Muzic RF. Performance evaluation of the Ingenuity TF PET/CT scanner with a focus on high count-rate conditions. Phys Med Biol. 2014;59:3843–3859. 24955921 10.1088/0031-9155/59/14/3843 PMC4457340

[24] Sprute K Kramer V Koerber SA. Diagnostic accuracy of ^18^F-PSMA-1007 PET/CT imaging for lymph node staging of prostate carcinoma in primary and biochemical recurrence. J Nucl Med. 2021;62:208–213. 32817141 10.2967/jnumed.120.246363 PMC8679593

[25] NDA 214793/piflufolastat F18 (PYLARIFY): multi-disciplinary review and evaluation. Food and Drug Administration website. https://www.accessdata.fda.gov/drugsatfda_docs/nda/2021/214793Orig1s000MultidisciplineR.pdf. Published May 26, 2021. Accessed March 5, 2025.

[26] Landis JR Koch GG. The measurement of observer agreement for categorical data. Biometrics. 1977;33:159–174. 843571

[27] Trabulsi EJ Rumble RB Jadvar H. Optimum imaging strategies for advanced prostate cancer: ASCO guideline. J Clin Oncol. 2020;38:1963–1996. 31940221 10.1200/JCO.19.02757

[28] Mattei A Fuechsel FG Bhatta Dhar N. The template of the primary lymphatic landing sites of the prostate should be revisited: results of a multimodality mapping study. Eur Urol. 2008;53:118–125. 17709171 10.1016/j.eururo.2007.07.035

